# Weldability and Mechanical Properties of Pure Copper Foils Welded by Blue Diode Laser

**DOI:** 10.3390/ma17092140

**Published:** 2024-05-02

**Authors:** Tim Pasang, Shumpei Fujio, Pai-Chen Lin, Yuan Tao, Mao Sudo, Travis Kuendig, Yuji Sato, Masahiro Tsukamoto

**Affiliations:** 1 Department of Engineering Design, Manufacturing and Management System, Western Michigan University, Kalamazoo, MI 49008-5200, USA; 2 Graduate School of Engineering, Osaka University, 2-1 Yamadaoka, Osaka 567-0871, Japan; fujio.shumpei.awx@ecs.osaka-u.ac.jp (S.F.); sudo.mao.25m@ecs.osaka-u.ac.nz (M.S.); 3 AIM-HI, National Chung Cheng University, No. 168, Section 1, Daxue Road, Minxiong, Chiayi 62102, Taiwan; imepcl@ccu.edu.tw; 4 Department of Mechanical Engineering, AUT University, Auckland 1010, New Zealand; yuan.tao@aut.ac.nz; 5 Department of Manufacturing, Mechanical Engineering and Technology, Oregon Institute of Technology, Klamath Falls, OR 97601, USA; travis.kuendig@oit.edu; 6 Joining and Welding Research Institute, Osaka University, 11-1 Mihogaoka, Osaka 567-0047, Japan; sato.yuji.jwri@osaka-u.ac.jp (Y.S.); tukamoto@osaka-u.ac.jp (M.T.)

**Keywords:** weldability, blue diode laser, pure copper, bead-on-plate, mechanical properties

## Abstract

The need to manufacture components out of copper is significantly increasing, particularly in the solar technology, semiconductor, and electric vehicle sectors. In the past few decades, infrared laser (IR) and green laser (GL) have been the primary technologies used to address this demand, especially for small or thin components. However, with the increased demand for energy saving, alternative joint techniques such as blue diode laser (BDL) are being actively explored. In this paper, bead-on-plate welding experiments on 0.2 mm thick pure copper samples employing a BDL are presented. Two sets of parameters were carefully selected in this investigation, namely Cu-1: Power (P) = 200 W; Speed (s) = 1 mm/s; and angle = 0°, and Cu-2: P = 200 W; s = 5 mm/s; and angle = 10°. The results from both sets of parameters produced defect-free full penetration welds. Hardness test results indicated relatively softer weld zones compared with the base metal. Tensile test samples fractured in the weld zones. Overall, the samples welded with Cu-1 parameters showed better mechanical properties, such as strength and elongation, than those welded with the Cu-2 parameters. The tensile strength and elongation obtained from Cu-1 were marginally lower than those of the unwelded pure copper. The outcomes from this research provide an alternative welding technique that is able to produce reliable, strong, and precise joints, particularly for small and thin components, which can be very challenging to produce.

## 1. Introduction

Copper is known to have attractive properties, such as high electrical conductivity and high thermal conductivity, which makes it a highly suitable material for application in renewable and solar energy technologies and in the electrical and electronic industries, including in semiconductors and electric vehicles, as well as many other niche applications. In solar technology, copper forms the backbone of heat exchangers, wiring, and cabling. In semiconductors, copper is used mainly as an interconnector between the elements of integrated circuits (ICs). In electric vehicles, many of the components, such as the coil in the motor, the batteries, the inverters, and the wiring, are either made of copper or contain a fairly significant amount of copper. Electric vehicles use as much as four times the amount of copper compared with a conventional car (also known as an internal combustion vehicle (ICE)). A significant number of parts deployed in these applications require reliable joints, which can be achieved through soldering, brazing, and welding. However, in general, welding provides the strongest joints, while soldering gives the weakest joints. Traditional fusion welding methods, such as gas tungsten arc welding (GTAW or TIG), gas metal arc welding (GMAW or MIG), shielded metal arc welding (SMAW), and plasma arc welding (PAW) are commonly used to weld copper and its alloys [1]. However, where smaller and thinner components are required, both GTAW and GMAW face challenges due to the size of their weld guns, their electrodes, and their degrees of precision and efficiency. This makes welding with laser more favorable since it provides flexibility and can be fully automated. The most common welding laser technology used to weld metals including copper has arguably been infrared (IR), which has a wavelength of around 1000 nm. However, there are a few challenges that need to be overcome before high quality and strong weld joints can be achieved with this technology. Copper can only absorb around 5% of the light emitted from the infrared laser; therefore, a considerable amount of energy is wasted [2]. High thermal conductivity is another limiting factor in copper welding, where heat that comes from the laser gun dissipates quickly across the whole sample. This may lead to incomplete fusion or lack of fusion.

To overcome these challenges, a few methods have been explored and reported on, such as (i) increasing the laser power, (ii) increasing the peak intensity, (iii) increasing the absorption by using a laser with shorter wavelengths, e.g., green laser, (iv) using hybrid methods, such as a combination of infrared and green laser or a combination of infrared and blue laser, and (v) applying a coating technique to improve the level of absorptivity of copper [3,4,5,6,7,8,9,10,11,12,13,14,15,16,17,18,19,20,21,22,23]. For example, Miyagi and Zhang performed spot welding of pure copper using a high-power disk laser [4]. They reported an increase in penetration depth and bead width with laser power. However, as the welding speed increased, the penetration depth and bead width decreased. The authors also reported that spatter and the formation of surface voids can be reduced significantly with high laser power and a welding speed of around 167 mm/s. A different approach was reported by Chen at al., who coated the copper surfaces of samples with nano-composite materials. They were able to successfully laser weld the samples without significantly reducing the properties [5].

The use of green laser technology has become increasingly more popular since it is suitable for copper welding due to its higher absorptivity, i.e., ≥5 times that of infrared laser light. Copper can absorb up to 50% of the light emitted by green laser. Engler et al. compared the use of IR with a wavelength of 1030 nm and green laser with a wavelength of 515 nm on copper samples with a thickness of 0.5 mm [8]. Full penetration was achieved when the power used was 600 W for IR and 200 W for the green laser. Hess et al. [9] conducted laser welding on copper alloy by combining the low power green laser spot (532 nm) and infrared laser spot (1064 nm), which resulted in a significant penetration. With IR power of 500 W, the depth was around 50 µm. When green laser was employed (P = 70 W), the depth increased to around 110 µm. Finally, when IR (P = 430 W) was used in combination with green laser (P = 70 W), a penetration depth of around 300 µm was achieved. All experiments were conducted with a speed of 25 m/min. Zhang et al. [10] conducted fiber laser experiments on pure copper with a thickness of 1.5 mm. The authors managed to obtain full penetration with power of 2.6 kW and a speed of 1 m/min. However, the ideal parameters, where the authors produced the best results, were a power level of 4 kW and a speed of 7 m/min. Beck et al. reported the use of green laser (wavelength around 515 nm) to perform micro-welding. With a power of 3000 W and speed of around 250 mm/s, they managed to achieve a penetration depth of up to 1.07 mm [11]. Heider et al. reported that the weld quality of copper can be positively affected by the modulation of laser power [12]. Furthermore, they also reported that penetration depth, as well as a smooth weld seam surface, could be obtained by modulation. Rüttimann et al. performed spot welding on 0.3 mm thick pure copper, in which they employed a mix of infrared (1000 nm wavelength) and green (532 nm wavelength) laser [13]. The authors claimed that they significantly increased the reproducibility, reliability, and efficiency of the spot welding, particularly for electronic and medical devices. Chung et al. investigated the weldability of copper with a disc laser at wavelength λ = 515 nm (green laser). They reported that the process duration was half of that if processed with λ = 1064 (infrared), due to the ability of copper to absorb more laser with the shorter wavelength [14]. Haubold et al. employed a high-power disc laser with a green wavelength with a power range from 100 W up to 1000 W. With a power of 100 W, no visible weld seam was observed. Penetration was observed when the power was higher than 450 W. Good penetration was observed, i.e., up to 270 µm, when a power of 700 W was employed at a speed of 60 mm/s [15]. 

With the ongoing demands for energy saving and higher productivity while maintaining the quality and efficiency of products, the development of blue laser has improved the ability to process highly reflective and highly thermal conductive metal through welding or additive manufacturing. Blue laser is a relatively new technology and has attracted interests both in research institutions and manufacturing industries. Its development and applications have been described by various authors [22,23,24,25,26,27,28,29,30,31,32,33,34,35,36,37,38,39,40]. Pelaprat et al. described the introduction of industry-based blue diode laser machines by NUBURU (Centennial, CO 80112, USA), known as AO-150 and AO-500. They claimed that the machines were capable of delivering the power, brightness, and high absorption level required for industrial applications [22]. Tsukamoto et al. described the development process in Japan including green and blue diode laser technologies [23]. Suwa et al. developed BLUE IMPACT, a newly developed blue diode laser which was used for processing copper foils and melting copper powder. The intention of this newly developed laser technology was for material processing. The authors suggested that a new BDL with a power of 100 W and a core diameter of 100 μm can improve the manufacturing quality of electronic and automotive components made of copper [24]. Hummel et al. [25] conducted a feasibility study by conducting micro-welding of copper with thickness ranging from 150 μm to 1 mm using a diode laser with a power of 150 W and wavelength of 450 nm. Both butt and lap joints were satisfactorily achieved up to a thickness of 500 μm. Zediker et al. managed to weld pure copper with a blue laser using a power of 500 W and a spot size of 215 μm. The authors claimed that neither porosity nor spatter was found [26]. In another study, Zediker et al. performed bead-on-plate welding on copper with a power of 600 W and a spot size diameter of 200 μm. According to the authors, the laser was well-absorbed by the samples, and therefore, full penetration was achieved [27]. In a battery manufacturing related weld, Silva Sa et al. reported various successes performed using NUBURU on 30 and 40 layers of 10 µm thick copper foils [28]. Full penetration was achieved with a power of 500 W. Heine et al. carried out spot welding experiments with a blue diode laser with power between 500 and 1,750 W. They observed a very smooth transition between heat conduction and deep penetration welding when using the laser with blue wavelength. It resulted in deeper penetration, particularly at slower welding speed [29]. Britten et al. and Behringer et al. suggested the use of high-powered blue laser to further increase the efficiency of metal processing methods such as welding, cutting, and soldering [30,31,32]. Furthermore, various authors have also presented their investigation outcomes after using blue diode laser for other potential applications, such as the welding of stainless steels, dissimilar welding, additive manufacturing, and cladding [33,34,35,36,37,38,39].

At Osaka University’s Joining and Welding Research Institute (JWRI), various works have been performed in this area, including developing the fundamentals of BDL technology capabilities, which include investigations on additive manufacturing and welding with BDL or hybrid technologies [40,41,42,43,44,45]. Pasang et al. conducted welding on 100 µm and 200 µm thick foils of commercially pure titanium using a BDL system with a power of up to 100 W. With a speed of 30 mm/s, they managed to produce very satisfactory welds [40]. Following the success with welding of less reflective CP Ti foils material using BDL, investigations on highly reflective metals were carried out. Takenaka et al. investigated the welding of 2 mm thick pure copper with a 1.5 kW machine using a blue diode laser. A few things were concluded from their experiments, including: (i) with a power of 1450 W and a speed of 25 mm/s, a penetration of up to 40% can be achieved, and (ii) with the same power and a speed of 100 mm/s, a spatter-free keyhole welding mode can also be achieved [41]. Shumpei et al. performed welding on copper wires by combining blue diode laser and infrared. They observed the process using a high-speed video camera and a thermal camera. It was suggested that welding efficiency of the pure copper wire increased with an increase in the intensity of the blue laser [42]. Other works, such as cladding and additive manufacturing, were also carried out. Sato et al. employed in situ X-ray to observe the formation mechanism of pure copper on stainless steel substrate (cladding). They suggested that the amount of bubble from the molten stainless steel and penetration depth depended on the laser input energy of the blue diode laser. Furthermore, they managed to deposit a dense pure copper layer on the stainless steel surface without pores [43]. Ono et al. developed a blue diode laser system specifically for metal deposition. It is known as the blue diode laser metal deposition system (B-LMD). The authors reported the success of producing a pure copper rod with a length (height) of 30 mm within 1.5 s [44].

In this paper, the results from the welding of pure copper foils with a thickness of 0.2 mm using the BDL welding method are presented. The absorptivity of the samples, the mechanical properties of both unwelded and welded samples, their microstructure, and their fracture mode were examined and reported. Digital image correlation (DIC), a post-processing feature, was also performed following the tensile testing to better understand the tensile stresses and strains.

## 2. Materials and Methods

In this research investigation, several experiments were conducted, including investigations on the laser absorptivity levels of copper, stainless steels, and other metals, bead-on-plate welding experiments, metallography and optical microscopy, mechanical testing (hardness and tensile), and scanning electron microscopy. 

### 2.1. Laser Absorptivity of Metals

A few articles have been published in relation to the level of laser absorptivity of metals [25,44,46]. However, understanding the absorptivity of the metals specifically used in this investigation was considered critical as they may have been slightly different in surface finish, which may have affected their brightness, along with their chemical composition and heat treatment properties, in contrast with those published earlier. The machine used to perform this task was a UV-2600 equipped with ISR-2600, made by Shimadzu Co. (Shimadzu Corporation, Kyoto, Japan)

### 2.2. Bead-on-Plate Welding with Blue Diode Laser

**Figure 1:**
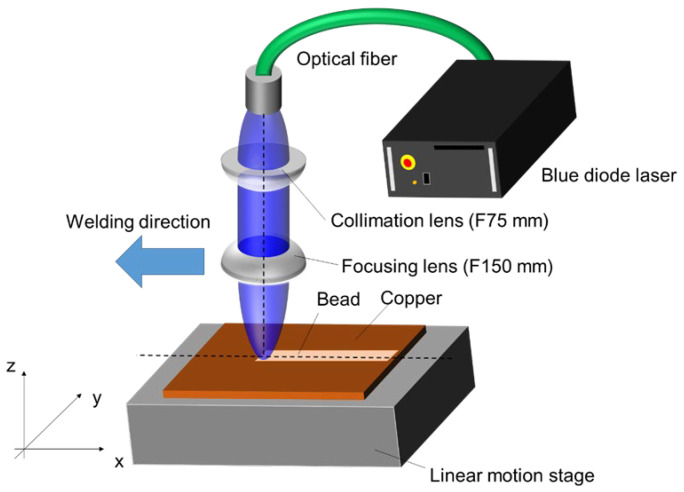
Schematic diagram of the welding process using blue diode laser (BDL) technology at JWRI, Osaka University.

Bead-on-plate welds were conducted on copper foils with a thickness of 0.2 mm. All welding experiments were performed at the Joining and Welding Research Institute (JWRI), Osaka University, Japan. Prior to welding, the foils were cleaned thoroughly with ethanol to ensure they were free from dirt, grease, and other surface contaminations. The Blue Diode Laser (BDL) system used had up to six diode laser modules. Each module had a power of around 20 W with a fiber core diameter of 100 µm and a wavelength of 450 nm. More details about the BDL system at JWRI have been provided by various authors [40,41,42,43,44]. Welding was performed without any filler metal (autogenous). The two carefully selected parameter sets used were Power (P) = 200 W, Speed (s) = 1 mm/s, and angle = 0° (aka Cu-1); and P = 200 W, s = 5 mm/s, and angle = 10° (aka Cu-2). The spot diameter of the BDL was set at 200 µm. Note that 0° indicates that the laser position is perpendicular to the weld surface. For comparison, an inclination angle of 10° was applied to minimize a possible direct reflection from the copper sample. The welding heat input was estimated to be around 200 J/mm and 40 J/mm, respectively. The welding process was performed under pure argon shielding gas with a flow of 25 L/min and a purity of up to 99.9%. A schematic diagram of the BDL welding used in this investigation is given in Figure 1. 

### 2.3. Metallography and Optical Microscopy

To investigate the weld quality and microstructures, a number of cross sections were prepared from the welded coupons. Standard metallography procedures were performed on these cross-section samples. They included grinding with 100 SiC paper and polishing up to 2400 SiC paper, followed by final polishing cloths with 0.3 µm diamond suspensions. The polished samples were then etched with a mixture of 50 mL distilled water + 50 mL Nitric acid reagent for 5–10 s [47]. An Olympus BX51M optical microscope (Olympus, Hachioji-shi, Tokyo 192-8507, Japan) was employed to examine the weld profiles, penetration, and the microstructural changes at the weld zones.

### 2.4. Mechanical Testing

#### 2.4.1. Hardness Testing

Hardness indentations were made across the base metal (BM), heat-affected zone (HAZ), and the fusion zone (FZ) on the metallographically-prepared samples using a Vickers machine (Leco AMH 55, St. Joseph, MI, USA). The load used was 50 gf with a dwell time of 10 s. Hardness profiles were obtained by plotting the hardness values vs. distance.

#### 2.4.2. Tensile Testing

**Figure 2:**
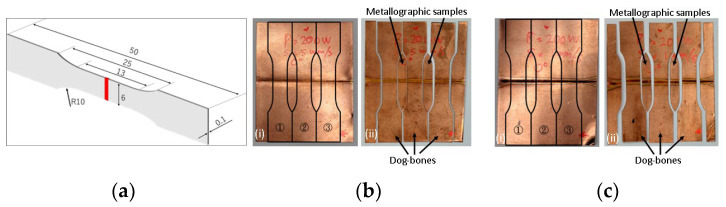
(**a**) Schematic diagram of the dog-bone sample with dimensions (in mm). Welded coupons (**i**) and dog-bone samples (**ii**) following BDL welding using the parameters of (**b**) P = 200 W, s = 5 mm/s 10°, and (**c**) P = 200 W, s = 1 mm/s 0°. Note: (**a** and **i**) sample drawings, and (**ii**) dog-bones and metallographic samples following the wire cutting.

Dog-bone samples were cut from the coupons using an EDM wire cutting machine. The drawing and dimensions of the samples are shown in Figure 2. A minimum of three samples were tested for each welding condition. The samples were tested using the Shimadzu Universal Testing Machine model AG-IS (Shimadzu, Kyoto, Japan) with a crosshead speed of 0.07 mm/s. The machine used was equipped with a digital image correlation (DIC) system with two 5.0 Megapixel CCD cameras (Correlated Solutions, Irmo, SC, USA), which provided a non-contact measurement. The VIC-3D system (Correlated Solutions, Irmo, SC, USA) was used to analyze the information provided in the DIC as part of the post-processing data analysis. The system was capable of measuring the overall strain and strain distribution as well as the local strains including, for example, at the fracture locations. For this to happen, uniform black spots were sprayed on the sample surface, which was painted white. The black spots were used as reference points; they were traced by the cameras [40,47]. 

#### 2.4.3. Fracture Surface Examination

Following tensile testing, the fractured samples were cleaned using ethanol in ultrasonic cleaner for 10 min. The fracture surfaces were then examined using a scanning electron microscope (SEM), Hitachi SU70 (Hitachi High-Technologies Corporation, Minato-ku, Tokyo, Japan).

## 3. Results and Discussions

### 3.1. Laser Absorptivity of Metals

**Figure 3:**
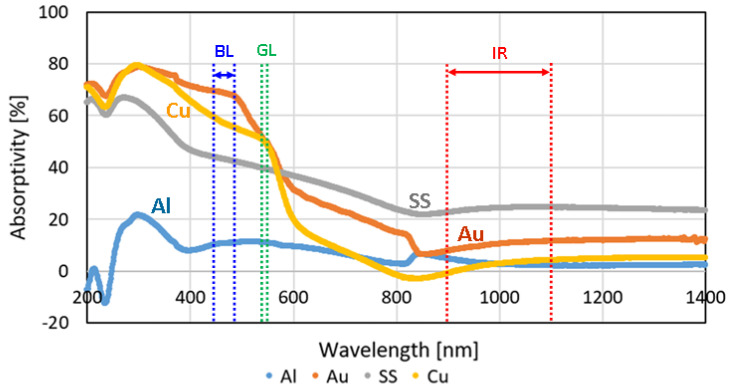
The absorptivity level of various metals (pure Al, pure Cu, pure Au, and SS316L) on a range of wavelengths using the UV-2600 equipped with ISR-2600 Shimadzu Co. (Shimadzu Corporation, Kyoto, Japan). The approximate wavelengths of infrared (IR), green laser (GL), and blue laser (BL) are indicated.

Figure 3 shows the results of the absorptivity experiment conducted at the JWRI Lab on various metals. The approximate wavelengths of infrared (IR), green laser (GL) and blue laser (BL) are also indicated, i.e., 900–1150 nm, 532 nm and 440–488 nm, respectively. It can be seen that the level of absorptivity of copper using IR laser is very low, i.e., around 5%. However, with green laser, the absorptivity is increased to around 50%. Furthermore, with blue diode laser (BDL), the level of absorptivity is significantly increased further to around 60% (Figure 3). These results are consistent with what was reported earlier by Hummel et al. [25] and Ono et al. [44].

### 3.2. Optical Micrographs

**Figure 4:**
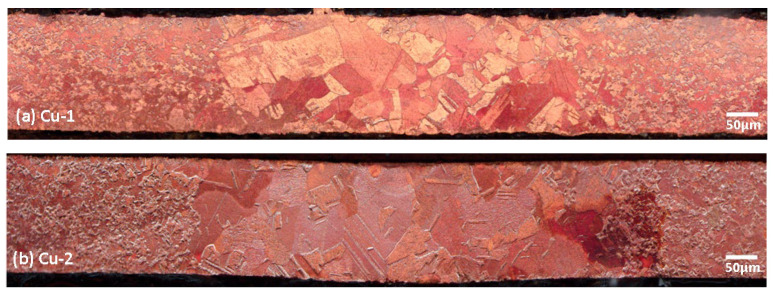
Weld profiles of samples welded with (**a**) Cu-1 and (**b**) Cu-2 parameters.

From Figure 4, it can be seen that with both welding parameters, full penetration was achieved. The weld zones were in a “square shape” instead of a typical V-shape or hourglass shape. This indicates a 2D heat flow for a thin sample, given the ratio of 1:2 between the spot size and the thickness of the sample. The high thermal conductivity of copper may also have contributed to this weld shape. It was also noticed that the width of the weld zones was different. The width of the weld zones of Cu-1 and Cu-2 were measured to be around 590–620 µm and 720–760 µm, respectively. With the angle of 0° (Cu-1 samples), the laser was directed perpendicular to the sample, while for Cu-2 sample, the angle of laser of 10° increased the size of the laser spot. This explains why Cu-2 had a wider weld zone. It is worth noting that the HAZ locations and width and the centerline were not obvious. This could be due to the ability of the blue laser to focus on a very narrow area and thus minimize the possible formation of a heat-affected zone (HAZ).

Grain shapes and sizes changed dramatically in the weld zones. The original grain shapes appeared to be equiaxed. The average grain size ranged from 5 to 20 µm in the BM area. However, grain size in the weld zones was significantly larger, i.e., up to around 100 µm. Many twin grains were also evident in the weld zone area.

### 3.3. Hardness and Tensile

The hardness profiles of the samples from both welding processes were identical, keeping in mind that the weld zone of Cu-2 sample was slightly wider (Figure 4). The BM area had higher hardness, i.e., around 75 HV, compared with both HAZ and FZ, with the latter being the softest, i.e., between 55 and 63 HV. As comparison, Zhang et al. [10] reported a hardness drop from 90–100 HV in the base metal down to 60–70 HV in the weld zone. Chen at al. also reported that the hardness of the welded samples was slightly reduced to around 74–88 HV from the original/unwelded hardness of around 89 HV [5]. Several plausible explanations may be associated with this phenomenon: (i) the grain structure changed from elongated/pancake-shaped (due to rolling) in the BM to equiaxed in both HAZ and FZ; or (ii) there were reduced dislocation numbers in the HAZ and FZ compared with the BM [5,10]. 

**Figure 5:**
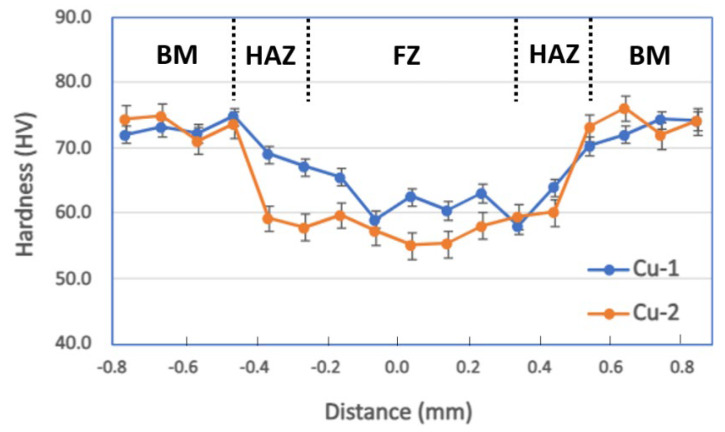
Hardness profiles of both samples, showing low hardness in the weld zones compared with the base metal. HAZ and FZ lines are indicative as they are not very clear on the optical micrographs.

Following tensile testing, hardness tests were conducted near the fracture area increased to 110–125 HV, while hardness at the BM was increased to 95–105 HV (Figure 5). This clearly shows the effect of strain hardening or work hardening on copper [48,49,50,51]. This is also obvious given the significant increase in hardness near the fracture location area, which had experienced higher strain compared with the area further from the fracture area.

**Figure 6:**
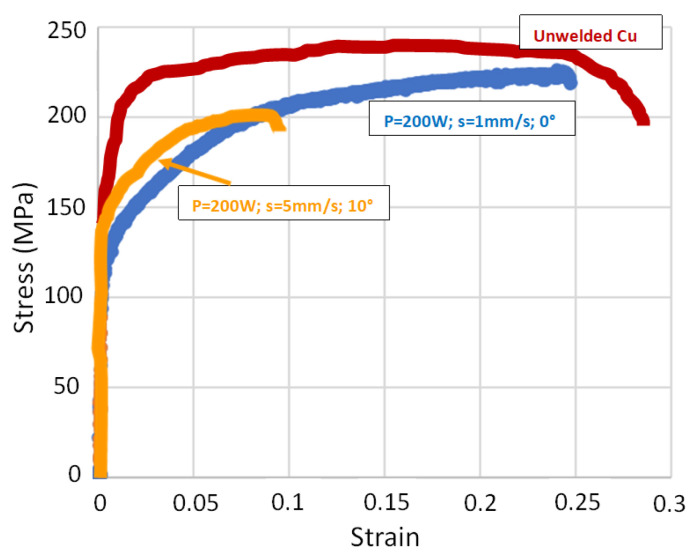
Stress–strain diagrams from tensile testing representing Cu-1, Cu-2, and unwelded samples.

**Figure 7:**
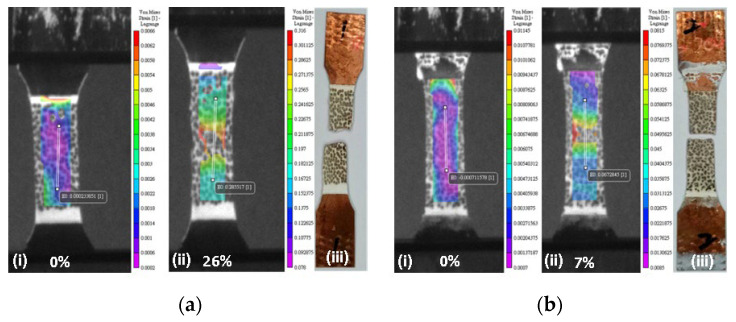
Images from digital image correlation (DIC) of welded Cu showing (**a**) P = 200 W; s = 1 mm/s; 0°, and (**b**) P = 200 W; s = 5 mm/s; 10°. Note: (**i**) is the initial stage, (**ii**) prior to fracture, and (**iii**) are the fractured samples.

The Cu-1 sample showed higher strength and elongation compared with sample Cu-2 (Table 1 and Figure 6). Comparing the mechanical properties of the unwelded sample, both the strength and elongation of the Cu-1 sample were around 7% and 20% below the unwelded sample, respectively. Furthermore, the strength and elongation of the Cu-2 sample were reduced by 15% and 80%, respectively. The images captured by the camera on the DIC systems are presented in Figure 7(i,ii). Figure 7(i) shows the initial stage for the two samples, while Figure 7(ii) represents the elongations prior to fracture, and Figure 7(iii) shows the fractured samples following tensile tests. In addition, from the DIC results, the elongation in the weld zone was measured to be around 28–32% for Cu-1 and 8–10% for Cu-2. The ability to withstand deformation locally at the weld zone (measured by DIC in terms of elongation) in the Cu-1 samples was within the elongation of the unwelded samples.

For comparison, Zhang et al. [10] reported full penetration of 1.5 mm thick pure copper with a power of 2.6 kW and a speed of 1 m/min. The best result was obtained with a power of 4 kW and speed of 7 m/min, under which they managed to obtain a strength of welded samples of around 200 MPa with 4% elongation. The unwelded samples had a strength of 250 MPa and 25% elongation. The results from these welded samples are comparable to our current results with Cu-2 parameters, but significantly lower than our Cu-1 results. Another study performed by Chen et al. on 0.5 mm thick pure copper reported an average tensile strength of 165 MPa for samples without nano-composite coating, and around 175 MPa for coated samples [5]. The strength of the unwelded samples was around 190 MPa. These values indicated a reduction in strength of around 13% for the uncoated sample and 8% for the coated sample as compared to the original strength. 

### 3.4. Fractography

**Figure 8:**
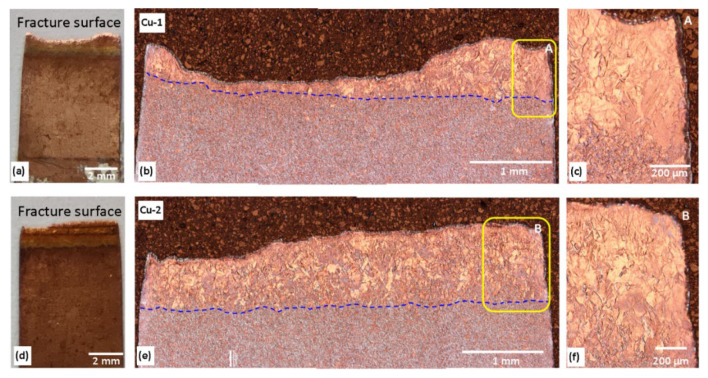
Macrographs and optical micrographs showing fractured tensile samples. Cu-1 sample fractured in the FZ and, probably, along the HAZ area (**a**–**c**), while the Cu-2 sample fractured in the FZ area only (**d**–**f**). The blue lines indicate weld zone/BM boundary. Insets: higher magnification micrographs on areas A and B.

Following tensile tests, the fractured samples were examined using both optical microscopy and scanning electron microscopy. Figure 8a-f shows the optical macrographs and micrographs. The macrographs showed reduction of width was more evident on the Cu-1 compared with Cu-2 sample. Upon grinding, polishing, and etching, the Cu-1 sample fractured along both the FZ and, possibly, along the HAZ as well (Figure 8b). The Cu-2 sample, however, showed fractures only along the FZ area. The boundary that separated the weld zone and BM was a relatively straight line (Figure 8b,e), except for the left section of Cu-1 sample, due to higher strain on the sample prior to fracture (Figure 8b) compared with Cu-2 sample. Figure 8c,f show highly deformed grains, which are barely recognizable (compared with the grains at the weld zone on Figure 4). Hardness values in these areas doubled, i.e., from 55–63 HV to 110–125 HV, confirming the strain hardening effect.

**Figure 9:**
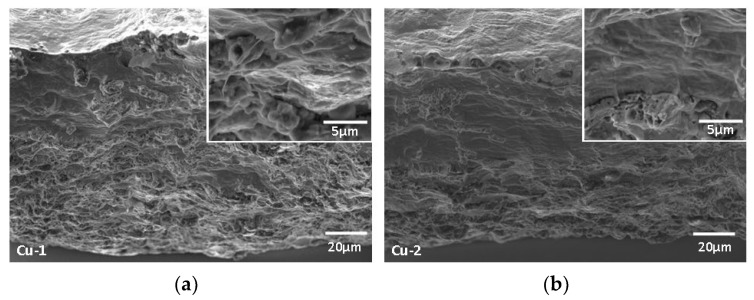
SEM images showing fracture surfaces of sample welded with (**a**) P = 200 W; s = 1 mm/s; 0°, and (**b**) P = 200 W; s = 5 mm/s; 10°. Insets: high magnification SEM shows dimples.

Fracture surface examinations with SEM showed that the fracture surfaces from the welds exhibited some differences (Figure 9). The fracture surface of the Cu-1 samples showed dimples on almost the entire surface compared with that of Cu-2 sample. This implies more ductility on sample Cu-1 compared with the Cu-2 sample, as also shown by the stress–strain diagram as well as the cross sectional reductions in Figure 8a,d. While the welded samples had a mixture of dimples and brittle fracture mechanisms, the unwelded samples had a completely ductile fracture mechanism.

## 4. Conclusions

An investigation on the weldability and mechanical properties of pure copper using blue diode laser technology was performed. The following points can be summarized:

1. The absorptivity examination of the samples used in this investigation confirmed their low absorption of infrared laser, i.e., around 6%. However, this increased to around 50% for the green laser, and to 60% for the blue diode laser.
2. A power of 200 W was proven to be sufficient for performing a bead-on-plate on 0.2 mm thick pure copper.
3. Two different angles and welding speeds were used, i.e., 0° at a speed of 1 mm/s (Cu-1) and 10° with a speed of 5 mm/s (Cu-2). The 0° angle could have potentially caused high reflection from the copper samples, since the laser faced the sample perpendicularly. However, this was found not to be an issue, given full penetration was achieved. Furthermore, the mechanical properties obtained from this parameter were very close to those of the unwelded copper samples.
4. The weld zones displayed a lower hardness (strength) due to the larger grain size compared with the base metal (BM). Given the nature of copper being a strain-hardened material, the loss of dislocations due to melting and solidification in the weld zones may also be one of the causes of this.
5. All samples fractured in the weld zones under tensile testing. The Cu-1 samples had mechanical properties slightly below the unwelded samples. Furthermore, hardness values around the fracture surface area doubled, which proved the strain hardening nature of copper.
6. DIC results showed localized deformation within the relatively softer weld zones. Further analysis of the DIC data indicated that the elongation in the weld zones area prior to fracture were within the range of elongation of unwelded samples.
7. Scanning electron micrograph images showed a microvoid coalescence (ductile) mechanism for samples from both groups. However, unlike Cu-2 samples, Cu-1 samples had almost the entire fracture covered with dimples, which strongly indicated a sound weld quality.
8. The outcomes from this investigation provide a reference for welding thin samples using BDL in, for example, the electrical and electronics field.

## Figures and Tables

**Table 1:** Mechanical properties of unwelded and welded samples.

Samples	Parameters	Yield Strength (MPa)	Tensile Strength (MPa)	Elongation (%)
Unwelded	N/A	195^±5^	241^±3^	30^±4^
Cu-1	P = 200 W; s = 1 mm/s; 0°	150^±6^	225^±7^	24^±3^
Cu-2	P = 200 W; s = 5 mm/s; 10°	162^±5^	205^±6^	6^±3^

## Data Availability

Data are contained within the article.
